# Retraction: Prognostic impact of prognostic nutritional index on renal cell carcinoma: A meta-analysis of 7,629 patients

**DOI:** 10.1371/journal.pone.0273158

**Published:** 2022-08-11

**Authors:** The PLOS ONE Editors

After this article was published, similarities were noted between this article and submissions by other research groups which call into question the reliability and provenance of the reported results. In light of these issues, the *PLOS ONE* Editors retract this article [1].

HW did not agree with retraction. QP, LL, TL, and CL did not respond or could not be reached.
